# Bacterial Phylogenetic Reconstruction from Whole Genomes Is Robust to Recombination but Demographic Inference Is Not

**DOI:** 10.1128/mBio.02158-14

**Published:** 2014-11-25

**Authors:** Jessica Hedge, Daniel J. Wilson

**Affiliations:** ^a^ Nuffield Department of Medicine, University of Oxford, John Radcliffe Hospital, Oxford, United Kingdom; ^b^ Wellcome Trust Centre for Human Genetics, Oxford, United Kingdom

## Abstract

Phylogenetic inference in bacterial genomics is fundamental to understanding problems such as population history, antimicrobial resistance, and transmission dynamics. The field has been plagued by an apparent state of contradiction since the distorting effects of recombination on phylogeny were discovered more than a decade ago. Researchers persist with detailed phylogenetic analyses while simultaneously acknowledging that recombination seriously misleads inference of population dynamics and selection. Here we resolve this paradox by showing that phylogenetic tree topologies based on whole genomes robustly reconstruct the clonal frame topology but that branch lengths are badly skewed. Surprisingly, removing recombining sites can exacerbate branch length distortion caused by recombination.

## IMPORTANCE

Phylogenetic tree reconstruction is a popular approach for understanding the relatedness of bacteria in a population from differences in their genome sequences. However, bacteria frequently exchange regions of their genomes by a process called homologous recombination, which violates a fundamental assumption of phylogenetic methods. Since many researchers continue to use phylogenetics for recombining bacteria, it is important to understand how recombination affects the conclusions drawn from these analyses. We find that whole-genome sequences afford great accuracy in reconstructing evolutionary relationships despite concerns surrounding the presence of recombination, but the branch lengths of the phylogenetic tree are indeed badly distorted. Surprisingly, methods to reduce the impact of recombination on branch lengths can exacerbate the problem.

## Observation

Phylogenetic methods are powerful and widely used tools for reconstructing the ancestral history of pathogen populations. These methods have been used extensively in evolutionary contexts and are increasingly applied to bacterial populations in clinical settings for strain classification and outbreak detection (1). Such applications require accurate estimation of the phylogenetic tree, but this can be problematic for bacteria due to recombination, in which DNA is exchanged via transformation, transduction, or conjugation (2). In the early 2000s, several authors demonstrated that recombination distorts phylogenetic inference, leading to biased estimates of branch lengths, artifactual signals of population expansion (3), false inference of positive selection (4, 5), and unreliable reconstruction of the tree topology (6, 7). Recombination causes tree topology and branch lengths to change along the genome, preventing a single tree from adequately explaining the reticulated ancestry of recombining sequences.

With the advent of accessible whole-genome sequencing, phylogenetic approaches are increasingly being used to reconstruct the evolutionary history of bacterial populations from their genome sequences (1, 8). The prevalence of phylogenetic analyses despite their demonstrable problems raises difficult questions concerning the credibility of conclusions drawn from phylogenetic inference. The esthetic appeal of phylogenetic trees partly explains their continued popularity, but the lack of viable alternatives is also an important factor. Several sophisticated methods attempt to model reticulated ancestries, but their practical application has been limited by computational demands (9–14). However, we contend that phylogenetic approaches have endured because biologists have found they convey meaningful information about the structure and relatedness of bacterial populations that fits with other evidence.

Milkman and Bridges (15) introduced the concept of the clonal frame to describe the phylogeny of sites in the bacterial genome that have not experienced recombination. Since a bacterial recombination event typically affects only a fraction of the genome, continual assault by recombination throughout the genome would be required to obliterate the signal of the clonal frame. Despite the attention given to the effect of recombination on phylogenetic inference, investigation into the accuracy of topological reconstruction has been limited to analyses of single or concatenated gene sequences and small sample sizes (6, 16). Therefore, we reasoned that phylogenetic inference might be reliably recovering the signal of the clonal frame from bacterial genomes, which could explain the continued faith placed in phylogenetic inference despite the problem of recombination.

**FIG 1:**
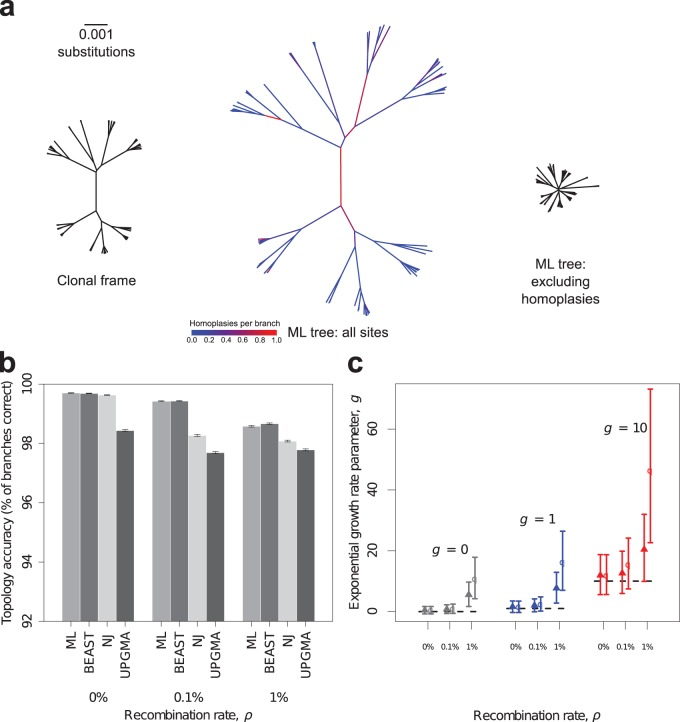
Effects of recombination in bacteria on phylogenetic tree topology and growth rate estimates. (a) The true clonal frame (left) and ML phylogenies constructed from all sites (center) and only nonhomoplastic sites (right) representing the evolutionary history of a population of 100 bacterial genomes of 1 million base pairs. The recombination rate (ρ) and substitution rate (θ) were fixed at 1%. The number of homoplasies per branch is shown for the center tree. (b) Estimates of branch accuracy for trees reconstructed using ML, BEAST, NJ, and UPGMA at three different values of ρ. The means and standard errors are based on 1,000 simulations of a demographic model of constant population size. (c) Mean posterior estimates of the exponential growth rate parameter (*g*) from BEAST, averaged over analyses of 1,000 simulated data sets. Data were simulated under a demographic model of constant population size (gray), low exponential growth (blue), and high exponential growth (red) and at three different values of ρ. Error bars represent the mean 95% confidence intervals. Estimates from analyses using either all sites in the sequence alignment (filled triangles) or only those sites without homoplasies (open circles) are plotted. Black dashed horizontal lines represent the true value of the exponential growth rate parameter used in the simulations.

We set out to test this idea through simulation. We simulated 1,000 populations of 100 bacterial genomes, each 1 Mb long with moderate mutation (substitution rate [θ] = 1%) under three scenarios: high, low, and no recombination (recombination rate [ρ] = 1%, 0.1%, and 0%, respectively). For each simulation, we recorded the clonal frame and estimated the phylogeny using neighbor joining (NJ) (17), unweighted-pair group method with arithmetic means (UPGMA) (18), maximum likelihood (ML) (19), and BEAST (20) (full details in Text S1 in the supplemental material). We quantified accuracy as the percentage of branches in the clonal frame correctly reconstructed. We found that the clonal frame topology was reconstructed remarkably accurately even when recombination was present (>97% [Fig. 1b]). Increasing ρ only modestly reduced accuracy, which appeared to be driven by the shorter branches (see Fig. S1 in the supplemental material). In a model of stable population size, branches nearer the tips tend to be shorter, whereas in an exponentially growing population, the tendency for tips to be shorter than deep branches is reduced, and at high growth rates, it is reversed (21). As such, branches closer to the root are less accurate at high recombination rates for exponentially growing populations (Fig. S2). In contrast, we found that bootstrap values (NJ, UPGMA, and ML) and posterior probabilities (BEAST) were upwardly biased by recombination (Fig. S3). Our results indicate that the accuracy of the tree topology decays progressively with increasing recombination rate. It follows that at very high recombination rates, it would no longer be sensible to pursue tree-based inference, although even at ρ = 8%, we found that topological accuracy remained high (93% based on 100 simulations with constant population size).

In contrast to the robustness of the phylogenetic topology, recombination gave rise to a spurious or inflated signal of demographic growth when we fitted a model of exponential growth using BEAST (Fig. 1c). In simulations under high, low, and no growth (exponential growth rate parameter [*g*] = 10, 1, and 0, respectively), growth rates were systematically overestimated, even though tree topology remained accurate (>98% for ρ = 0.1% and 1%; see Fig. S4 in the supplemental material).

Some authors have recommended the removal of recombining sites to ameliorate their detrimental effect on phylogenetic analysis, in particular the tendency for recombination to produce a spurious signal of exponential growth (22–24). Recombination generates various signatures including homoplasy, in which the same substitution is observed in different parts of the tree. Homoplasy can be generated by repeat and back mutation, but it also results from reshuffling diversity among ancestral lineages by recombination, so that excess homoplasy is indicative of levels of recombination sufficient to cause problems for phylogenetic inference (25). We investigated whether removing homoplastic sites improved the estimation of exponential growth rates by BEAST. We found that removing homoplasies actually exacerbated the spurious signal of demographic growth generated by recombination (Fig. 1c), because older recombination events were more likely to be detected as homoplasies. This led to preferential removal of substitutions from the deep branches of the tree, producing trees that appeared even more star-like (Fig. 1a). The magnitude of the effect increased with higher recombination rates, producing 95% confidence intervals that excluded the true growth rate. The number of homoplastic sites removed due to repeat and back mutation amounted to 0.2% of the genome and had a negligible effect on the estimation of growth rates (observed in the absence of recombination in Fig. 1c). We found that removal of homoplasies followed by reestimation of the phylogeny had limited effect on the accuracy of the topology itself (see Fig. S5 in the supplemental material).

In summary, our results show that the clonal frame topology is robustly reconstructed from bacterial whole genomes by phylogenetic methods even in the presence of recombination, but the branch lengths of the clonal frame are not. Removal of recombining sites exacerbates branch length distortion, because older events are easier to detect than young ones, meaning that phylogenetic-based demographic inference should still be viewed with caution in recombining species.

## SUPPLEMENTAL MATERIAL

Text S1 Supplemental methods. Additional details of the simulations, phylogenetic tree construction, calculation of tree accuracy, and identification of recombining sites are given. Download Text S1, PDF file, 0.1 MB

Figure S1 Branch accuracy for trees reconstructed using ML, BEAST, NJ, and UPGMA at three different values of the recombination rate (*ρ*) and growth rate (*g*). Branches are partitioned into three intervals according to their length, selected in an attempt to keep the number of branches within intervals the same (mean of 65.3 branches). The mean number of branches per interval for each method is displayed above each bar. Means and standard errors are based on analyses of 1,000 simulations under  a demographic model of constant population size (*g* = 0) (gray), low exponential growth (*g* = 1) (blue), and high exponential growth (*g* = 10) (red). Download Figure S1, PDF file, 0.05 MB

Figure S2 Branch accuracy for trees reconstructed using ML, BEAST, NJ, and UPGMA at three different values of the recombination rate (*ρ*) and growth rate (*g*). Branches are partitioned into three intervals according to the distance between the end of the branch and the root node. These intervals differed between growth rates in an attempt to keep the number of branches within intervals the same (mean of 65.3 branches). The mean number of branches per interval for each method is displayed above each bar. Means and standard errors are based on analyses of 1,000 simulations under a demographic model of constant population size (*g* = 0) (gray), low exponential growth (*g* = 1) (blue), and high exponential growth (*g* = 10) (red). Download Figure S2, PDF file, 0.04 MB

Figure S3 Accuracy of branches in estimated trees partitioned by the accuracy of either the bootstrap value or posterior probability support for each branch. Trees were reconstructed using ML, BEAST, NJ, and UPGMA at three different values of the recombination rate (*ρ*). The expected linear relationship between support and accuracy is plotted in blue. Means and standard errors are based on analyses of 1,000 simulations under a demographic model of constant population size. Download Figure S3, PDF file, 0.1 MB

Figure S4 Branch accuracy for trees reconstructed using ML, BEAST, NJ, and UPGMA at three different values of the recombination rate (*ρ*) and growth rate. Means and standard errors are based on analyses of 1,000 simulations under a demographic model of constant population size (*g* = 0) (gray), low exponential growth (*g* = 1) (blue), and high exponential growth (*g* = 10) (red). Download Figure S4, PDF file, 0.04 MB

Figure S5 Branch accuracy for trees reconstructed using ML, BEAST, NJ, and UPGMA from genome sequence alignments after the removal of homoplasies. Data were simulated under three different values of the recombination rate (*ρ*) and growth rate (*g*). Means and standard errors are based on analyses of 1,000 simulations under a demographic model of constant population size (*g* = 0) (gray), low exponential growth (*g* = 1) (blue), and high exponential growth (*g* = 10) (red). The accuracy of NJ trees is marginally greater than for those constructed from all sites (see Fig. S3 in the supplemental material), due to the alignment being enriched for sites supporting the clonal frame. However, the accuracy of UPGMA trees is lower after the removal of homoplasies when ρ = 1%. Download Figure S5, PDF file, 0.03 MB

## References

[1] Didelot X Bowden R Wilson DJ Peto TEA Crook DW 2012 Transforming clinical microbiology with bacterial genome sequencing. Nat. Rev. Genet. 13:601–612. 10.1038/nrg3226. 22868263 PMC5049685

[2] Smith JM Smith NH O’Rourke M Spratt BG 1993 How clonal are bacteria? Proc. Natl. Acad. Sci. U. S. A. 90:4384–4388. 10.1073/pnas.90.10.4384. 8506277 PMC46515

[3] Schierup MH Hein J 2000 Consequences of recombination on traditional phylogenetic analysis. Genetics 156:879–891. 11014833 10.1093/genetics/156.2.879 PMC1461297

[4] Anisimova M Nielsen R Yang Z 2003 Effect of recombination on the accuracy of the likelihood method for detecting positive selection at amino acid sites. Genetics 164:1229–1236. 12871927 10.1093/genetics/164.3.1229 PMC1462615

[5] Shriner D Nickle DC Jensen MA Mullins JI 2003 Potential impact of recombination on sitewise approaches for detecting positive natural selection. Genet. Res. 81:115–121. 10.1017/S0016672303006128. 12872913

[6] Posada D Crandall KA 2002 The effect of recombination on the accuracy of phylogeny estimation. J. Mol. Evol. 54:396–402. 10.1007/s00239-001-0034-9. 11847565

[7] Rannala B Yang Z 2008 Phylogenetic inference using whole genomes. Annu. Rev. Genomics Hum. Genet. 9:217–231. 10.1146/annurev.genom.9.081307.164407. 18767964

[8] Wilson DJ 2012 Insights from genomics into bacterial pathogen populations. PLoS Pathog. 8:e1002874. 10.1371/journal.ppat.1002874. 22969423 PMC3435253

[9] McVean GAT Cardin NJ 2005 Approximating the coalescent with recombination. Philos. Trans. R. Soc. Lond. B Biol. Sci. 360:1387–1393. 10.1098/rstb.2005.1673. 16048782 PMC1569517

[10] Huson DH Bryant D 2006 Application of phylogenetic networks in evolutionary studies. Mol. Biol. Evol. 23:254–267. 10.1093/molbev/msj030. 16221896

[11] Bloomquist EW Suchard MA 2010 Unifying vertical and nonvertical evolution: a stochastic ARG-based framework. Syst. Biol. 59:27–41. 10.1093/sysbio/syp076. 20525618 PMC2909786

[12] Didelot X Lawson D Darling A Falush D 2010 Inference of homologous recombination in bacteria using whole-genome sequences. Genetics 186:1435–1449. 10.1534/genetics.110.120121. 20923983 PMC2998322

[13] Kuhner MK Yamato J Felsenstein J 2000 Maximum likelihood estimation of recombination rates from population data. Genetics 156:1393–1401. 11063710 10.1093/genetics/156.3.1393 PMC1461317

[14] Fearnhead P Donnelly P 2001 Estimating recombination rates from population genetic data. Genetics 159:1299–1318. 11729171 10.1093/genetics/159.3.1299 PMC1461855

[15] Milkman R Bridges MM. 1990 Molecular evolution of the Escherichia coli chromosome. III. Clonal frames. Genetics 126:505-527. 1979037 10.1093/genetics/126.3.505 PMC1204208

[16] Kubatko LS Degnan JH 2007 Inconsistency of phylogenetic estimates from concatenated data under coalescence. Syst. Biol. 56:17–24. 10.1080/10635150601146041. 17366134

[17] Saitou N Nei M 1987 The neighbor-joining method: a new method for reconstructing phylogenetic trees. Mol. Biol. Evol. 4:406–425. 3447015 10.1093/oxfordjournals.molbev.a040454

[18] Sneath PHA Sokal RR 1973 Numerical taxonomy: the principles and practice of numerical classification. A series of books in biology. W. H. Freeman & Co, San Francisco, CA.

[19] Felsenstein J 1981 Evolutionary trees from DNA sequences: a maximum likelihood approach. J. Mol. Evol. 17:368–376. 10.1007/BF01734359. 7288891

[20] Drummond AJ Rambaut A 2007 BEAST: Bayesian evolutionary analysis by sampling trees. BMC Evol. Biol. 7:214. 10.1186/1471-2148-7-214. 17996036 PMC2247476

[21] Slatkin M Hudson RR 1991 Pairwise comparisons of mitochondrial DNA sequences in stable and exponentially growing populations. Genetics 129:555–562. 1743491 10.1093/genetics/129.2.555 PMC1204643

[22] Morelli G Song Y Mazzoni CJ Eppinger M Roumagnac P Wagner DM Feldkamp M Kusecek B Vogler AJ Li Y Cui Y Thomson NR Jombart T Leblois R Lichtner P Rahalison L Petersen JM Balloux F Keim P Wirth T Ravel J Yang R Carniel E Achtman M 2010 Yersinia pestis genome sequencing identifies patterns of global phylogenetic diversity. Nat. Genet. 42:1140–1143. 10.1038/ng.705. 21037571 PMC2999892

[23] Dress AWM Flamm C Fritzsch G Grünewald S Kruspe M Prohaska SJ Stadler PF 2008 Noisy: identification of problematic columns in multiple sequence alignments. Algor. Mol. Biol. 3:7. 10.1186/1748-7188-3-7. PMC2464588 18577231

[24] Hornstra HM Priestley RA Georgia SM Kachur S Birdsell DN Hilsabeck R Gates LT Samuel JE Heinzen RA Kersh GJ Keim P Massung RF Pearson T 2011 Rapid typing of Coxiella burnetii. PLoS One 6:e26201. 10.1371/journal.pone.0026201. 22073151 PMC3206805

[25] Maynard Smith J Smith NH 1998 Detecting recombination from gene trees. Mol. Biol. Evol. 15:590–599. 10.1093/oxfordjournals.molbev.a025960. 9580989

