# Supplementary material for: Bacterial Phylogenetic Reconstruction from Whole Genomes Is Robust to Recombination but Demographic Inference Is Not
Source: mBio. 2014 Nov 25;5(6):e02158-14. doi: 10.1128/mBio.02158-14 (PMC4251999; doi:10.1128/mBio.02158-14)
Supplement: Text S1 — Supplemental methods. Additional details of the simulations, phylogenetic tree construction, calculation of tree accuracy, and identification of recombining sites are given. Download [file mbo006142084s1.pdf]

Supplementary Methods

1. Simulating recombining bacterial populations

Multiple sequence alignments and their corresponding clonal frames were jointly simulated 1000 times under a neutral coalescent model with bacterial recombination and a Jukes-Cantor model of nucleotide substitution using SimMLST (1). Each population comprised 100 genome sequences of 1 million base pairs in length, which we partitioned into 1000 loci of equal length for computational efficiency. The population-scaled mutation rate, $\theta = 2N_e u$ (where N_e is the effective population size and u the mutation rate per site per generation) was fixed to 1%, a typical value for many bacterial species (2) and the average recombination tract length to 500 base pairs, similar to estimates from several species (Fearnhead et al. 2005; Jolley et al. 2005; Kennemann et al. 2011; Everitt et al. 2014). The population-scaled recombination rate, $\rho = 2N_e r$ (where r is the rate of initiation of recombination per site per generation), was fixed to 0%, 0.1% or 1%. At ρ = 1%, recombination events are initiated as often as mutation events, but the overall effect of recombination on the substitution process, known as r/m, is greater than that of mutation (r/m = 5) because each recombination event affects many sites. Therefore the range of recombination rates investigated encompasses those seen in the majority of bacteria, with the notable exception of extremely promiscuous species such as *Helicobacter pylori*, *Streptococcus pneumoniae* and *Salmonella enterica* (7).

Since recombination has been shown to produce spurious signals of exponential growth in phylogenies (8), we also studied the effect of recombination on branch accuracy in populations with different rates of growth. The growth rate parameter, $g = N_e m$ (where m is the exponential growth rate per generation), was investigated across a range of values of g = 0, 1 and 10, covering both low and high growth rates. Since exponential growth reduces the total number of substitutions across the tree, data simulated with the same value of θ under higher growth rates will comprise fewer substitutions. To make a fairer comparison we scaled θ in order to maintain the same expected number of mutations per tree across all demographic models. This was achieved by simulating the ratio of the average tree length constructed under a model of constant population size and one of exponential growth (g = 1 and g = 10).

2. Phylogenetic tree construction

Phylogenetic trees were constructed for each simulated dataset using the distance-based neighbor joining (NJ) and UPGMA methods, maximum likelihood (ML), and BEAST, which is a Bayesian inference method. In each analysis, a Jukes-Cantor (JC) model of nucleotide substitution was used (9). ML trees were constructed using PhyML with the following command line arguments: -m HKY85 -v 0 -t 1 -f 0.25,0.25,0.25,0.25 -c 1 -s BEST -b 100 (10). Bayesian phylogenetic trees were constructed in BEAST v.1.7.5 (Bayesian Evolutionary Analysis by Sampling Trees), using a strict molecular clock (uniform prior) and exponential growth model (populations size fixed to 1.0) (XML available on request) (11, 12).

Since ML was shown to reconstruct the most accurate tree topology, this tree was used as a starting tree in each BEAST analysis. This required midpoint rooting of the tree and rescaling of terminal branches, such that tip heights were zero (a requirement of starting trees for exponential growth models in BEAST). Two independent Markov chain Monte Carlo (MCMC) chains were run for 10 million steps each, which provided sufficient mixing and convergence to the stationary distribution. Parameters and trees from both runs were sampled every 1000 steps and combined using LogCombiner. Model parameters were summarized using LogAnalyser. NJ and UPGMA trees were constructed using the APE and phangorn libraries in R respectively (13, 14).

Bootstrapping of ML trees was performed in PhyML and of NJ and UPGMA trees in R, using 100 replicates in each case. Posterior probabilities of branches in BEAST trees were calculated by constructing the maximum clade credibility (MCC) tree for each distribution in TreeAnnotator (11, 12) .

3. Calculating tree topology accuracy of estimated trees

The accuracy of tree topology was calculated using the Robinson-Foulds Symmetric Difference metric (15) between the clonal frame and reconstructed tree. This was used to obtain the proportion of branches in the clonal frame correctly reconstructed in the estimated tree, i.e. accuracy = (total number of branches – (Symmetric Distance/2))/total number of branches. Unrooted trees were used for each comparison and the accuracy for each model was averaged

over 1000 simulated datasets. The accuracy of each posterior distribution of BEAST trees was quantified as the average accuracy across 1000 trees in the distribution, which did not differ from those estimated using the MCC tree.

In order to investigate how accuracy of branches varied by branch length, each branch in a tree was assigned to one of three intervals based on its length. Intervals were defined so that the number of branches per interval was approximately equal (mean: 65.3). The average accuracy of branches within each interval is plotted in Figure S2.

The accuracy of bootstrap values (ML, NJ, UPGMA) and posterior probabilities (BEAST) was measured as the mean proportion of correctly estimated branches within each of ten intervals of branch support value.

4. Removal of homoplasious sites from sequence alignment

Recombination events within a population can give rise to homoplasies across the phylogenetic tree. In order to remove all substitutions arising from recombination, all sites at which a homoplasy had occurred were removed from the alignment. Homoplasies were identified using maximum likelihood ancestral state reconstruction (16) to reconstruct the sequences at internal nodes in the ML tree and then counting the number of times each substitution arose on the tree. A homoplasious site is defined as one at which the minimum number of substitutions needed to explain the observed number of alleles is exceeded.

Supplementary References

1. **Didelot X, Lawson D, Falush D**. 2009. SimMLST: Simulation of multi-locus sequence typing data under a neutral model. Bioinformatics **25**:1442–1444.

2. **Pérez-Losada M, Browne EB, Madsen A, Wirth T, Viscidi RP, Crandall KA**. 2006. Population genetics of microbial pathogens estimated from multilocus sequence typing (MLST) data. Infect. Genet. Evol. **6**:97–112.

3. **Jolley KA, Wilson DJ, Kriz P, McVean G, Maiden MCJ**. 2005. The influence of mutation, recombination, population history, and selection on patterns of genetic diversity in Neisseria meningitidis. Mol. Biol. Evol. **22**:562–569.

4. **Fearnhead P, Smith NGC, Barrigas M, Fox A, French N**. 2005. Analysis of recombination in Campylobacter jejuni from MLST population data. J. Mol. Evol. **61**:333–340.

5. **Kennemann L, Didelot X, Aebischer T, Kuhn S, Drescher B, Droege M, Reinhardt R, Correa P, Meyer TF, Josenhans C, Falush D, Suerbaum S**. 2011. Helicobacter pylori genome evolution during human infection. Proc. Natl. Acad. Sci. U. S. A. **108**:5033–5038.

6. **Everitt RG, Didelot X, Batty EM, Miller RR, Knox K, Young BC, Bowden R, Auton A, Votintseva A, Larner-Svensson H, Charlesworth J, Golubchik T, Ip CLC, Godwin H, Fung R, Peto TE a., Walker a. S, Crook DW, Wilson DJ**. 2014. Mobile elements drive recombination hotspots in the core genome of Staphylococcus aureus. Nat. Commun. **5**:3956.

7. **Vos M, Didelot X**. 2009. A comparison of homologous recombination rates in bacteria and archaea. ISME J. **3**:199–208.

8. **Schierup MH, Hein J**. 2000. Consequences of recombination on traditional phylogenetic analysis. Genetics **156**:879–891.

9. **Jukes TH, Cantor CR**. 1969. Evolution of protein molecules, p. 21–132. *In* In Mammalian protein metabolism, Vol. III (1969), pp. 21-132.

10. **Guindon S, Gascuel O**. 2003. A simple, fast, and accurate algorithm to estimate large phylogenies by maximum likelihood. Syst. Biol. **52**:696–704.

11. **Drummond AJ, Rambaut A**. 2007. BEAST: Bayesian evolutionary analysis by sampling trees. BMC Evol. Biol. **7**:214.

12. **Drummond AJ, Suchard MA, Xie D, Rambaut A**. 2012. Bayesian phylogenetics with BEAUti and the BEAST 1.7. Mol. Biol. Evol. **29**:1969–1973.

13. **Paradis E, Claude J, Strimmer K**. 2004. APE: Analyses of phylogenetics and evolution in R language. Bioinformatics **20**:289–290.

14. **Schliep KP**. 2011. phangorn: phylogenetic analysis in R. Bioinformatics **27**:592–593.

15. **Robinson DF, Foulds LR**. 1981. Comparison of phylogenetic trees. Math. Biosci.

16. **Pupko T, Pe'er I, Shamir R, Graur D**. 2000. A fast algorithm for joint reconstruction of ancestral amino acid sequences. Mol. Biol. Evol. **17**:890–896.
