# Supplementary figures and images for: Bacterial Phylogenetic Reconstruction from Whole Genomes Is Robust to Recombination but Demographic Inference Is Not
Source: mBio. 2014 Nov 25;5(6):e02158-14. doi: 10.1128/mBio.02158-14 (PMC4251999; doi:10.1128/mBio.02158-14)

# Figure S1

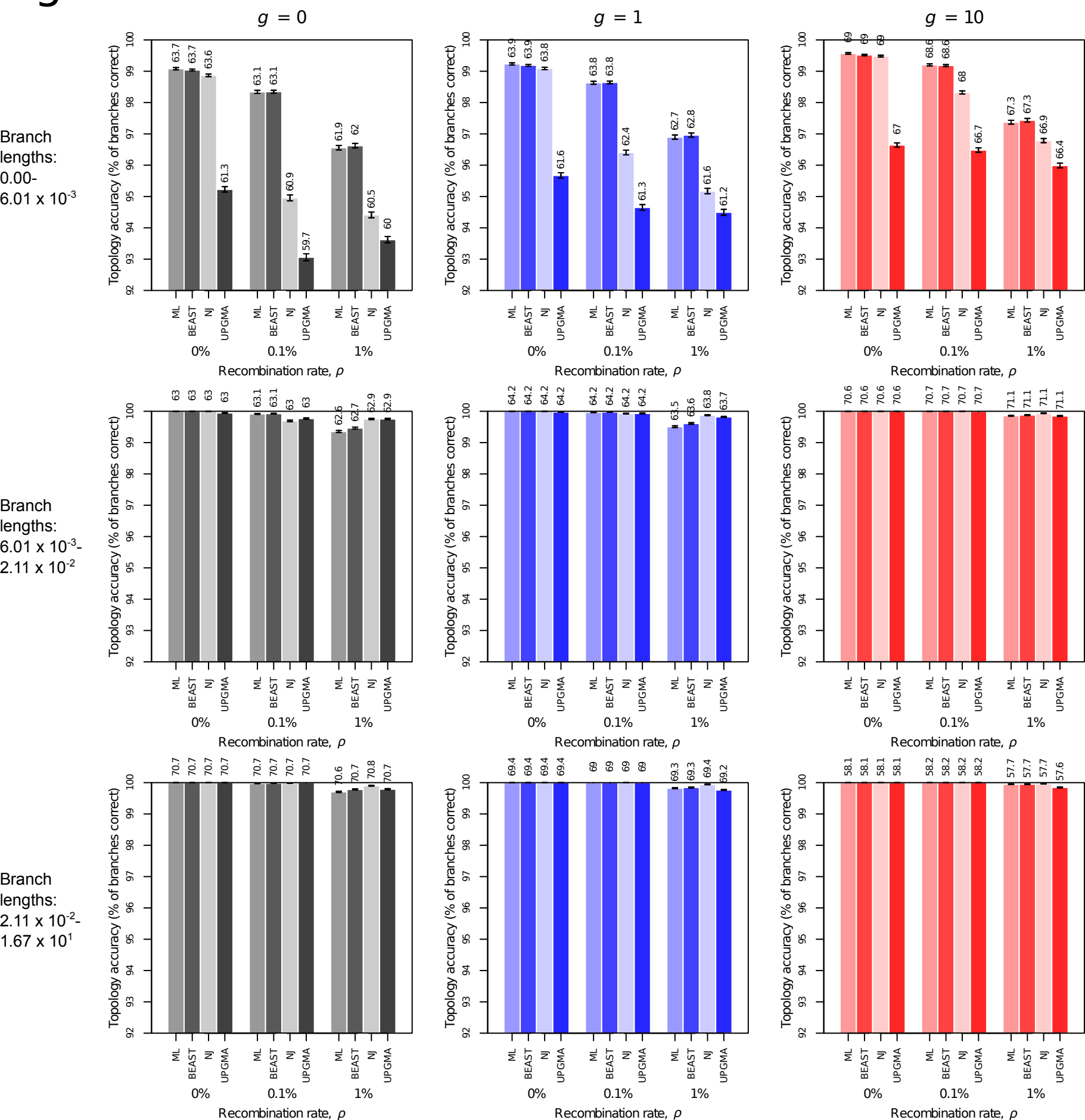

Supplement: Figure S1 — Branch accuracy for trees reconstructed using ML, BEAST, NJ, and UPGMA at three different values of the recombination rate (ρ) and growth rate (g). Branches are partitioned into three intervals according to their length, selected in an attempt to keep the number of branches within intervals the same (mean of 65.3 branches). The mean number of branches per interval for each method is displayed above each bar. Means and standard errors are based on analyses of 1,000 simulations under a demographic model of constant population size (g = 0) (gray), low exponential growth (g = 1) (blue), and high exponential growth (g = 10) (red). Download [file mbo006142084sf1.pdf]

# Figure S3

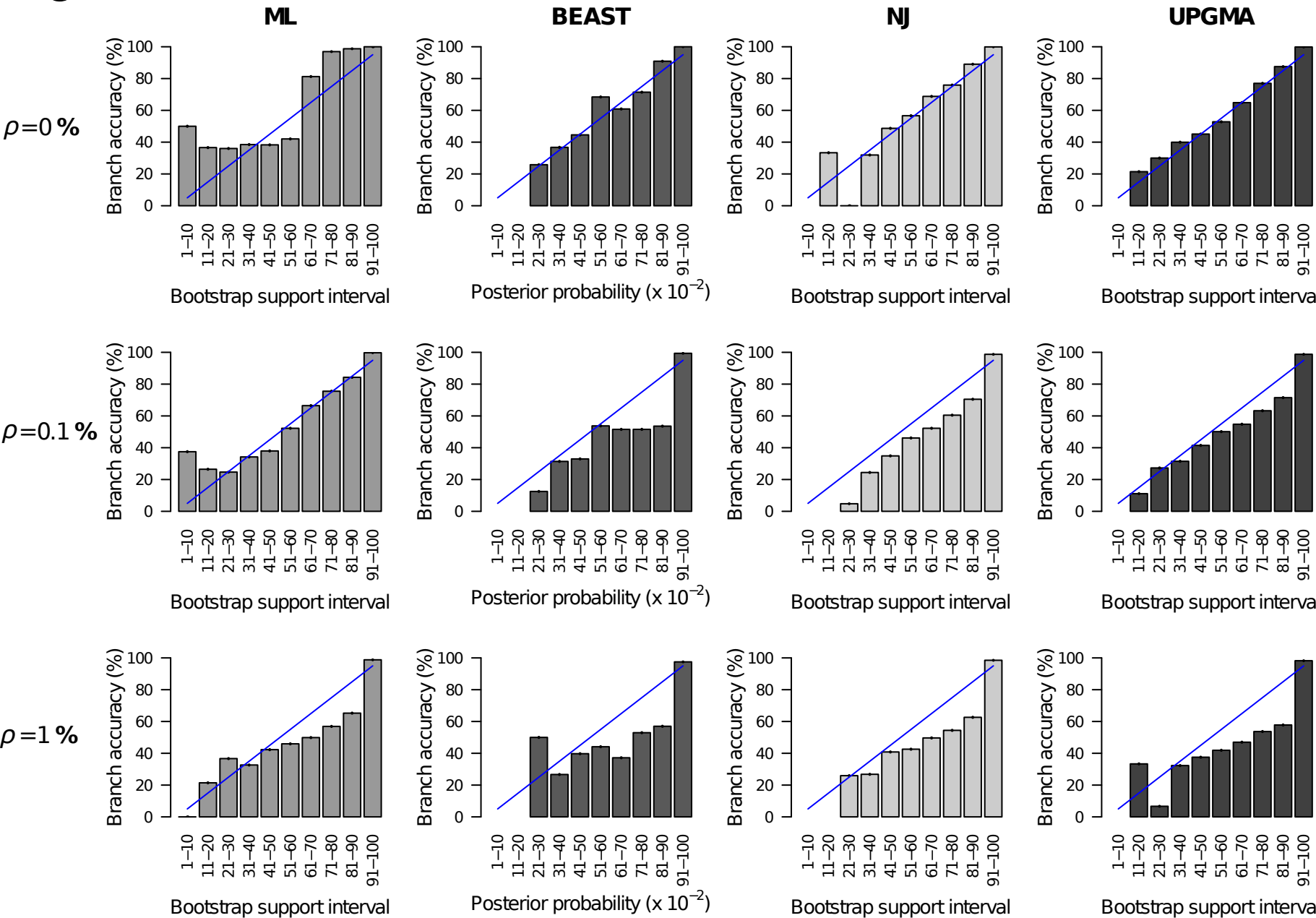

Supplement: Figure S3 — Accuracy of branches in estimated trees partitioned by the accuracy of either the bootstrap value or posterior probability support for each branch. Trees were reconstructed using ML, BEAST, NJ, and UPGMA at three different values of the recombination rate (ρ). The expected linear relationship between support and accuracy is plotted in blue. Means and standard errors are based on analyses of 1,000 simulations under a demographic model of constant population size. Download [file mbo006142084sf3.pdf]
