# Supplementary material for: Bacterial Phylogenetic Reconstruction from Whole Genomes Is Robust to Recombination but Demographic Inference Is Not
Source: mBio. 2014 Nov 25;5(6):e02158-14. doi: 10.1128/mBio.02158-14 (PMC4251999; doi:10.1128/mBio.02158-14)
Supplement: Figure S2 — Branch accuracy for trees reconstructed using ML, BEAST, NJ, and UPGMA at three different values of the recombination rate (ρ) and growth rate (g). Branches are partitioned into three intervals according to the distance between the end of the branch and the root node. These intervals differed between growth rates in an attempt to keep the number of branches within intervals the same (mean of 65.3 branches). The mean number of branches per interval for each method is displayed above each bar. Means and standard errors are based on analyses of 1,000 simulations under a demographic model of constant population size (g = 0) (gray), low exponential growth (g = 1) (blue), and high exponential growth (g = 10) (red). Download [file mbo006142084sf2.pdf]

Figure S2

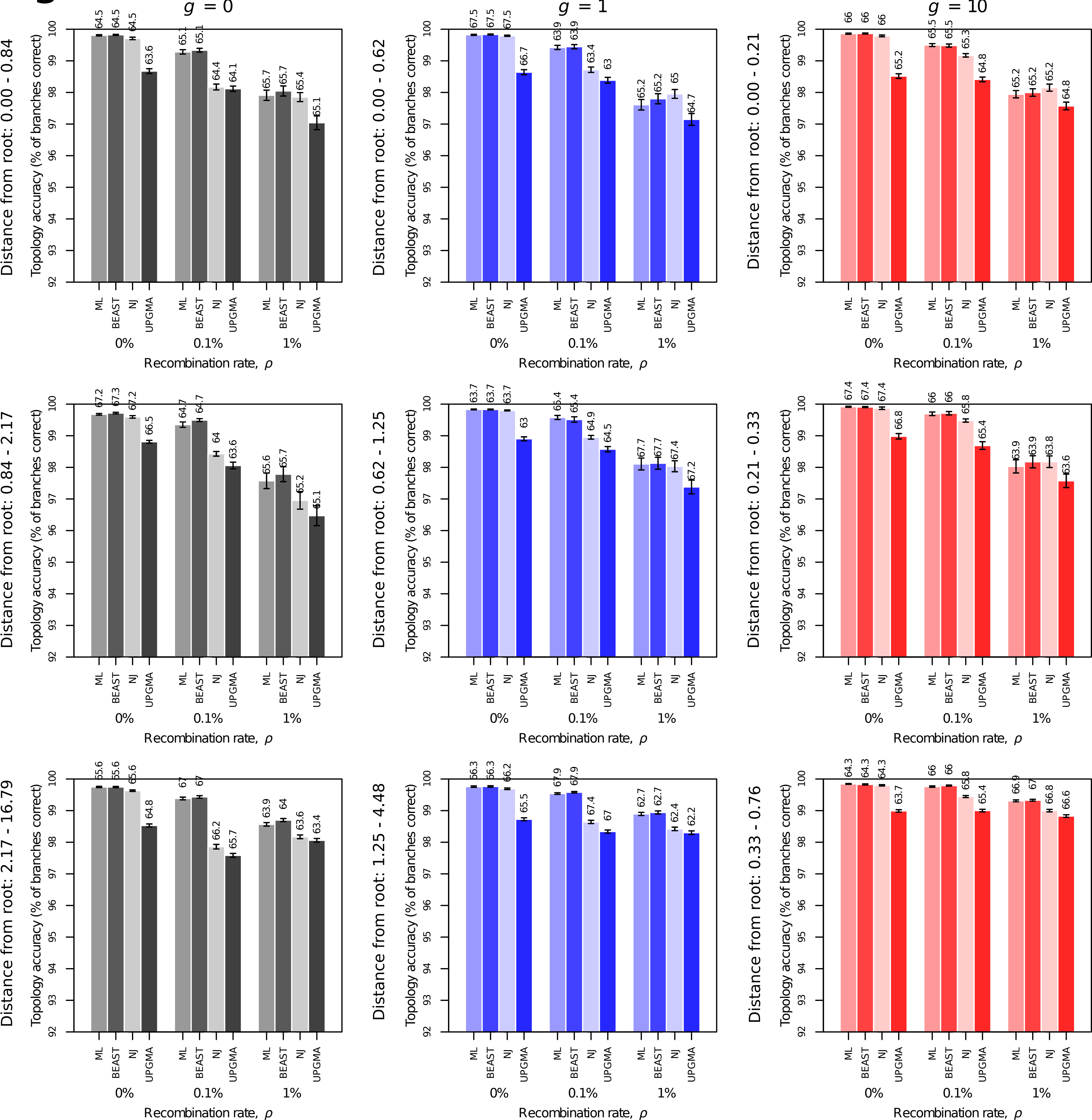

g = 0
g = 1
g = 10
Distance from root: 0.00 - 0.84
Distance from root: 0.00 - 0.62
Distance from root: 0.00 - 0.21
Distance from root: 0.84 - 2.17
Distance from root: 0.62 - 1.25
Distance from root: 0.21 - 0.33
Distance from root: 2.17 - 16.79
Distance from root: 1.25 - 4.48
Distance from root: 0.33 - 0.76
Topology accuracy (% of branches correct)
92 93 94 95 96 97 98 99 100
ML BEAST NJ UPGMA
0% 0.1% 1%
Recombination rate, ρ
64.5 64.5 64.5 63.6 65.1 65.1 64.4 64.1 65.7 65.7 65.4 65.1
67.5 67.5 67.5 66.7 63.9 63.9 63.4 63 65.2 65.2 65 64.7
66 66 66 65.2 65.5 65.5 65.3 64.8 65.2 65.2 65.2 64.8
67.2 67.3 67.2 66.5 64.7 64.7 64 63.6 65.6 65.7 65.2 65.1
63.7 63.7 63.7 63 65.4 65.4 64.9 64.5 67.7 67.7 67.4 67.2
67.4 67.4 67.4 66.8 66 66 65.8 65.4 63.9 63.9 63.8 63.6
65.6 65.6 65.6 64.8 67 67 66.2 65.7 63.9 64 63.6 63.4
66.3 66.3 66.2 65.5 67.9 67.9 67.4 67 62.7 62.7 62.4 62.2
64.3 64.3 64.3 63.7 66 66 65.8 65.4 66.9 67 66.8 66.6
