# Supplementary material for: Bacterial Phylogenetic Reconstruction from Whole Genomes Is Robust to Recombination but Demographic Inference Is Not
Source: mBio. 2014 Nov 25;5(6):e02158-14. doi: 10.1128/mBio.02158-14 (PMC4251999; doi:10.1128/mBio.02158-14)
Supplement: Figure S4 — Branch accuracy for trees reconstructed using ML, BEAST, NJ, and UPGMA at three different values of the recombination rate (ρ) and growth rate. Means and standard errors are based on analyses of 1,000 simulations under a demographic model of constant population size (g = 0) (gray), low exponential growth (g = 1) (blue), and high exponential growth (g = 10) (red). Download [file mbo006142084sf4.pdf]

Figure S4

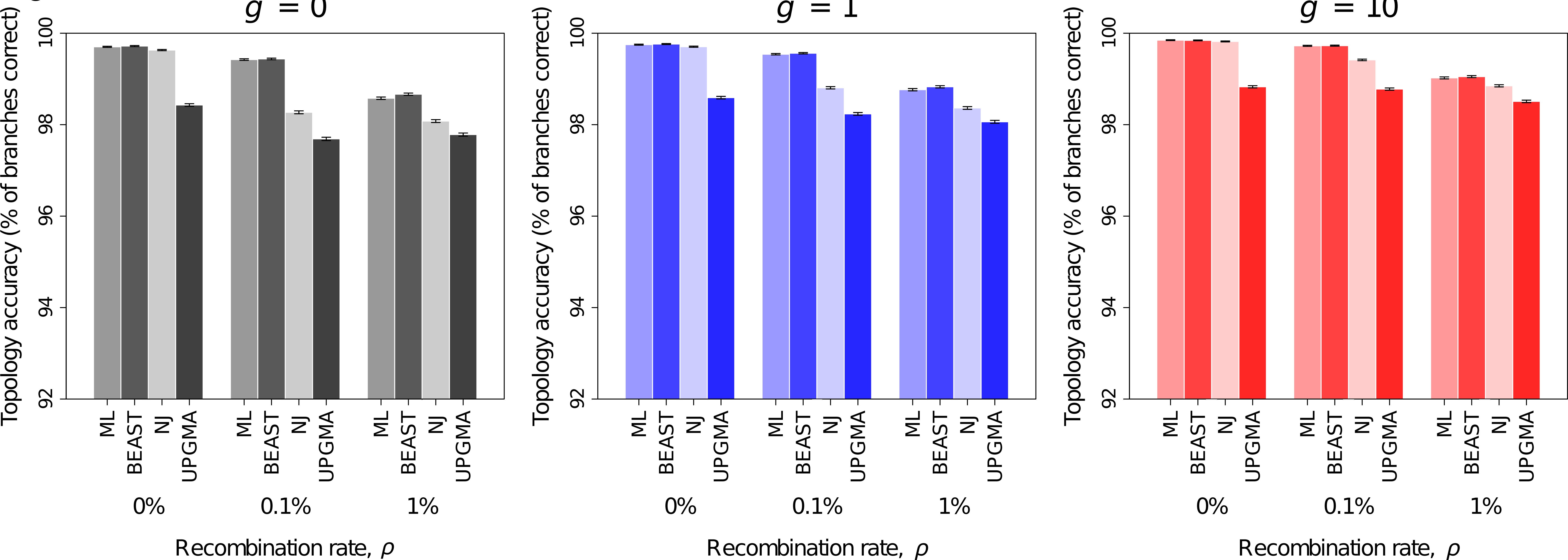

$g = 0$
$g = 1$
$g = 10$
Topology accuracy (% of branches correct)
100
98
96
94
92
ML
BEAST
NJ
UPGMA
0%
0.1%
1%
Recombination rate, ρ
