# Supplementary material for: Bacterial Phylogenetic Reconstruction from Whole Genomes Is Robust to Recombination but Demographic Inference Is Not
Source: mBio. 2014 Nov 25;5(6):e02158-14. doi: 10.1128/mBio.02158-14 (PMC4251999; doi:10.1128/mBio.02158-14)
Supplement: Figure S5 — Branch accuracy for trees reconstructed using ML, BEAST, NJ, and UPGMA from genome sequence alignments after the removal of homoplasies. Data were simulated under three different values of the recombination rate (ρ) and growth rate (g). Means and standard errors are based on analyses of 1,000 simulations under a demographic model of constant population size (g = 0) (gray), low exponential growth (g = 1) (blue), and high exponential growth (g = 10) (red). The accuracy of NJ trees is marginally greater than for those constructed from all sites (see Fig. S3 in the supplemental material), due to the alignment being enriched for sites supporting the clonal frame. However, the accuracy of UPGMA trees is lower after the removal of homoplasies when ρ = 1%. Download [file mbo006142084sf5.pdf]

Figure S5

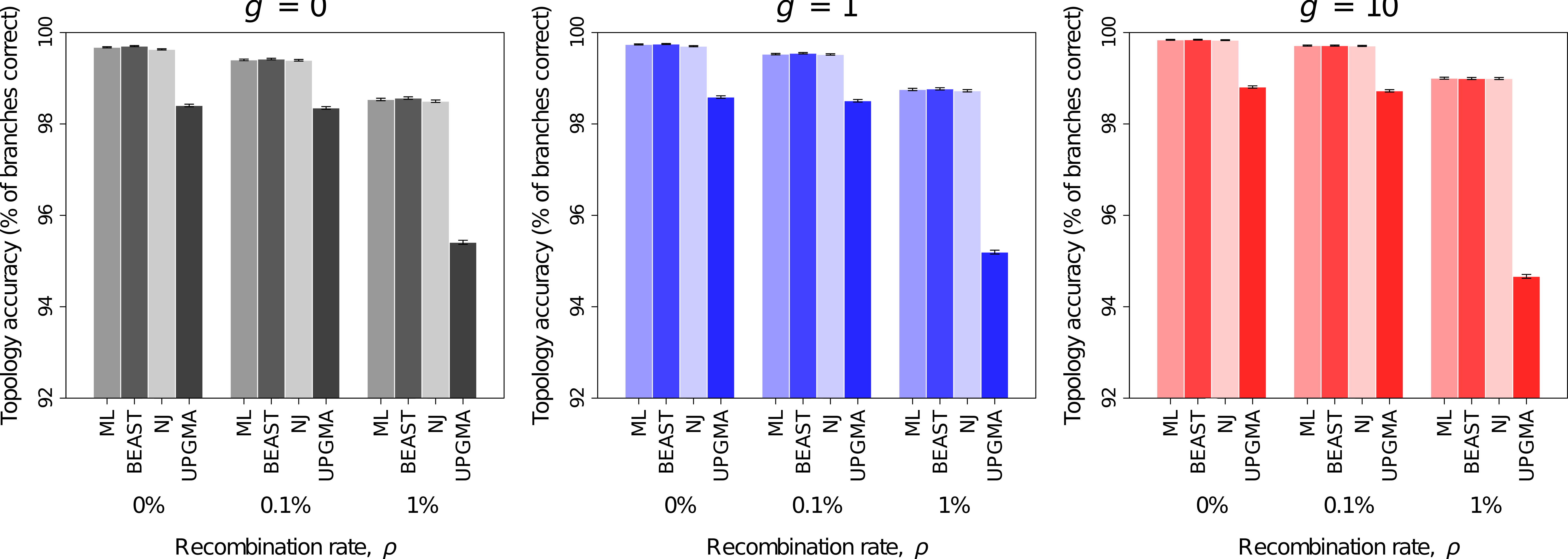

g = 0
g = 1
g = 10
Topology accuracy (% of branches correct)
100
98
96
94
92
ML
BEAST
NJ
UPGMA
0%
0.1%
1%
Recombination rate, ρ
